# Transcriptome dataset of omental and subcutaneous adipose tissues from gestational diabetes patients

**DOI:** 10.1038/s41597-022-01457-5

**Published:** 2022-06-17

**Authors:** David Salcedo-Tacuma, Leonardo Bonilla, Maria Cristina Geney Montes, Jorge Ernesto Niño Gonzalez, Sandra Milena Sanchez Gutierrez, Miguel Chirivi, G. Andres Contreras

**Affiliations:** 1 Department of Large Animal Clinical Sciences, College of Veterinary Medicine, Michigan State University, East Lansing, MI 48824 USA; 2 Hospital Universitario Clinica San Rafael, Bogota, Colombia

**Keywords:** Genetics research, Gene expression

## Abstract

Gestational diabetes (GD) is one of the most prevalent metabolic diseases in pregnant women worldwide. GD is a risk factor for adverse pregnancy outcomes, including macrosomia and preeclampsia. Given the multifactorial etiology and the complexity of its pathogenesis, GD requires advanced omics analyses to expand our understanding of the disease. Next generation RNA sequencing (RNA-seq) was used to evaluate the transcriptomic profile of subcutaneous and omental adipose tissues (AT) collected from patients with gestational diabetes and matched controls. Samples were harvested during cesarean delivery. Results show differences based on anatomical location and provide whole-transcriptome data for further exploration of gene expression patterns unique to GD patients.

## Abstract (summary)

**Table:** 

Measurement(s)	RNA sequencing
Technology Type(s)	Paired-End Sequencing
Factor Type(s)	Group • Adipose Tissue Site
Sample Characteristic - Organism	Homo sapiens
Sample Characteristic - Environment	city
Sample Characteristic - Location	Bogota, Colombia

## Background & Summary

Approximately 7% of all pregnant women develop gestational diabetes (GD) worldwide^1^. GD prevalence in the US is higher, with 9.2% of the pregnancies diagnosed every year^2^. Short and long-term maternal and child health complications prevalent in GD patients include macrosomia, preeclampsia, and type 2 diabetes^3^. Macrosomia in GD patients appears to be associated with peripheral insulin resistance and lipolysis dysregulation in adipose tissues (AT)^4^. The latter is defined as enhanced and protracted lipolysis that is not responsive to insulin’s anti-lipolytic actions leading to increased and sustained levels of free fatty acids in the blood^5,6^. In fact, circulating free fatty acids can better predict macrosomia in cases of GD and in those pregnancies complicated by obesity^6,7^. As a consequence, even in GD patients with adequate glucose control, the incidence of large for gestational age babies is high, reaching 30–50%^8,9^. Elucidating early triggers of AT insulin resistance and lipolysis dysregulation will minimize the incidence of maternal and neonatal complications in GD.

Using next generation RNA sequencing (RNA-seq), this study evaluated the whole transcriptome of subcutaneous (SC) and omental (OM) AT from patients with gestational diabetes (GD) and healthy matching controls collected during cesarean delivery (C-section). The inclusion of SC and OM supports the evaluation of AT site variations considering depot-specific differences in inflammatory and immune responses and insulin sensitivity^10,11^. Results show a strong separation of the transcriptomic profiles based on anatomical location and reveal specific RNA expression patterns unique to GD patients.

## Methods

### Ethics statement

This experiment was approved by Michigan State University and Hospital Universitario Clinica San Rafael institutional review boards (IRB). All patients provided written informed consent following the guidelines established by the ethics committee of Hospital Universitario Clinica San Rafael. Prior to sample preparation, all samples were anonymized by assigning a patient ID number.

### Patients and adipose tissue sampling

GD patients (n = 5) were recruited during the third trimester of gestation. Inclusion criteria were age = 18–45; gestational age at the moment of C-section = 37–41weeks; programmed C-section with fasting of at least 8 h; GD diagnosis during the second trimester of gestation based on blood biomarkers of dysfunctional carbohydrate metabolism. Matching controls (n = 5) had the same inclusion criteria except for GD diagnosis. Patients with multiple pregnancy, diabetes (Type I or II) diagnosis prior to pregnancy, hypertension, hypo or hyperthyroidism, autoimmune diseases, chronic diseases, and active tuberculosis were excluded. Table 1 presents a descriptive summary of demographics and blood biomarkers of the GD patients and controls.

**Table 1:** Age, body mass index (BMI), blood insulin, glucose, and HbA1C levels in gestational diabetes patients and controls at enrollment during the third trimester of gestation.

Patient ID	Age	Group	Pregestational BMI	Insulin (µU/mL)^1^	Glucose (mg/dL)^1^	HbA_1C_ (%)^1^	Glycemic Control
02	20	Control	23.43	7.60	67.2	5.2	—
07	28	Control	20.0	11.20	69.0	4.9	—
08	29	Control	21.0	5.20	74.0	4.4	—
14	29	Control	31.0	18.40	69.7	5.8	—
15	35	Control	27.0	8.00	77.4	5.5	—
03	27	Gestational Diabetes	28.0	9.70	69.3	5.4	—
04	22	Gestational Diabetes	35.7	16.40	87.1	5.5	Insulin^2^
09	32	Gestational Diabetes	33.0	8.00	74.0	5.3	Insulin
10	28	Gestational Diabetes	30.0	17.80	74.8	5.3	Insulin
11	37	Gestational Diabetes	26.0	17.20	73.0	5.3	Metformin^3^

^1^At the moment of blood collection, patients were fasted for at least 8 h.

^2^Insulin therapy included NPH and crystalline insulin 3 times per day preprandial.

^3^Metformin (850 mg/day).

AT samples from the SC and OM depots were collected during the C-Section. In brief, the SC samples were harvested from the incision area using a surgical scalpel. OM samples were collected from the surgical area using scissors and ligature at the omentum majus level. Both AT samples were flash-frozen and stored in liquid nitrogen until processing. Then, total RNA was extracted from OM and SC samples using Trizol and the Quick RNA MiniPrep kit (R1054; Zymo Research, Irving, CA, USA) that includes a DNase step to remove genomic DNA according to the manufacturer’s protocol.

## Data Records

Raw FASTQ data is available in the NCBI Gene Expression Omnibus (GEO) NCBI GSE188799^12^. Raw read count matrix was also deposited in the NCBI Gene Expression Omnibus (GEO) under accession number GSE188799^12^. Processed read count matrix and DEGs found in patients with gestational diabetes are available in (Supplemental Table 1^13^).

## Technical Validation

Purity, concentration, and integrity of mRNA were checked using a NanoDrop 1000 spectrophotometer (Thermo Scientific, Wilmington, DE, USA) and an Agilent Bioanalyzer 2100 system (Agilent Technologies, Santa Clara, CA, USA). All samples had a 260:280 nm ratio between 1.9 and 2.1 and RNA integrity number ≥ 7 (Table 2). At least 1 µg of each sample was used for NGS.

**Table 2:** RNA quantification and quality control variables including 260/280 ratio and RNA integrity number (RIN#).

Sample Name	Species	Adipose Tissue Site	Group	RNA Concentration ng/uL	260/280 Ratio	RIN #
2 SC	Human	Subcutaneous	Control	29	1.91	8.9
3 SC	Human	Subcutaneous	Gestational Diabetes	58.7	1.99	8.5
4 SC	Human	Subcutaneous	Gestational Diabetes	42	2.02	6.8
7 SC	Human	Subcutaneous	Control	20.8	2.16	7.6
8 SC	Human	Subcutaneous	Control	35.8	2	7.3
9 SC	Human	Subcutaneous	Gestational Diabetes	43.9	2.01	6.1
10 SC	Human	Subcutaneous	Gestational Diabetes	27.5	2.02	7.7
11 SC	Human	Subcutaneous	Gestational Diabetes	24.8	2.02	9
14 SC	Human	Subcutaneous	Control	98.2	1.98	7.2
15 SC	Human	Subcutaneous	Control	25.9	2.08	8.3
2 OM	Human	Omental	Control	135.2	1.98	8.6
3 OM	Human	Omental	Gestational Diabetes	104	1.98	7.8
4 OM	Human	Omental	Gestational Diabetes	124.2	1.99	7.7
7 OM	Human	Omental	Control	174.9	2.04	7.9
8 OM	Human	Omental	Control	502	1.96	7.1
9 OM	Human	Omental	Gestational Diabetes	119.5	1.99	7.7
10 OM	Human	Omental	Gestational Diabetes	43.4	1.93	8.9
11 OM	Human	Omental	Gestational Diabetes	74.3	1.97	8.9
14 OM	Human	Omental	Control	128.5	1.99	7.5
15 OM	Human	Omental	Control	64.1	1.93	8.3

### RNA sequencing

All RNA-seq was performed at the Beijing Genomics Institute [BGI, Shenzhen/Hong Kong, China (www.genomics.cn)] and paired-end sequencing (100 bp) was performed on the DNBSEQ platform. BGI’s process includes filtration and exclusion of reads with excessively high levels of unknown base N, adaptor contamination and low-quality reads with a score below 15. On average, 4.5 million adapter sequences were filtered, and the average size of clean reads was 4.46 Gb per sample (range 4.43–4.48 Gb). The ratio of clean reads was 93,7% (Table 3). RNA raw sequencing data was obtained in fastq-files from BGI and subsequent data processing and quality control was performed with FastQC v0.11.8^14^ (www.bioinformatics.babraham.ac.uk/projects/fastqc/) by the authors.

**Table 3:** Total reads and clean reads obtained from next-generation sequencing of omental and subcutaneous adipose tissue depots collected during C-Section from gestational diabetes patients and controls.

Sample Name	Adipose Tissue Site	Group	Total Raw Reads (M)	Total Clean Reads (M)	Total Clean Bases (Gb)	Clean Reads Ratio (%)
2OM	Omental	Control	47.48	44.68	4.47	94.1
2SC	Subcutaneous	Control	47.48	44.82	4.48	94.41
3OM	Omental	Gestational Diabetes	47.48	44.57	4.46	93.88
3SC	Subcutaneous	Gestational Diabetes	47.48	44.71	4.47	94.17
4OM	Omental	Gestational Diabetes	47.48	44.57	4.46	93.89
4SC	Subcutaneous	Gestational Diabetes	47.48	44.78	4.48	94.31
7OM	Omental	Control	47.48	44.62	4.46	93.99
7SC	Subcutaneous	Control	47.48	44.45	4.45	93.64
8OM	Omental	Control	47.48	44.6	4.46	93.95
8SC	Subcutaneous	Control	47.48	44.26	4.43	93.22
9OM	Omental	Gestational Diabetes	47.48	44.42	4.44	93.55
9SC	Subcutaneous	Gestational Diabetes	47.48	44.52	4.45	93.77
10OM	Omental	Gestational Diabetes	47.48	44.28	4.43	93.27
10SC	Subcutaneous	Gestational Diabetes	47.48	44.46	4.45	93.65
11OM	Omental	Gestational Diabetes	47.48	44.57	4.46	93.89
11SC	Subcutaneous	Gestational Diabetes	47.48	44.54	4.45	93.82
14OM	Omental	Control	47.48	44.72	4.47	94.19
14SC	Subcutaneous	Control	47.48	44.7	4.47	94.15
15OM	Omental	Control	49.97	45.43	4.54	90.91
15SC	Subcutaneous	Control	47.48	44.62	4.46	93.99

### Quality assessment of total RNA and RNA-Seq data

**Fig. 1:**
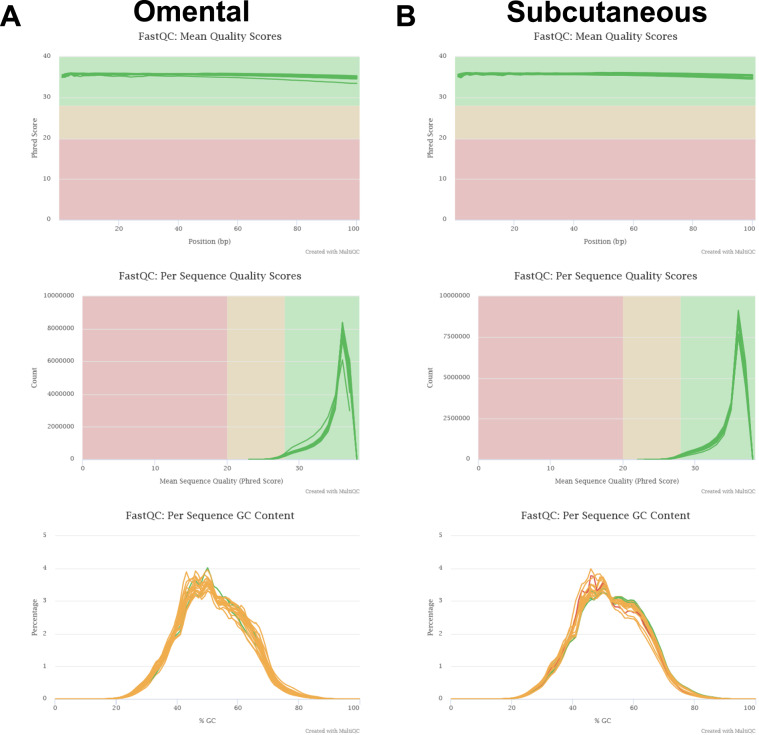
Evaluation of sequence quality scores in raw FASTQ data. The quality of FASTQ files was estimated using FastQC and summary plots for different samples were mapped on MultiQC. All 40 FASTQ files were assessed, and plots for GC content, mean quality per-base and per-sequence quality in terms of Phred score are presented. In (**A**) Results for Omental samples and (**B**) results for Subcutaneous samples.

Data quality of the raw RNA-seq reads from FastQC was compiled using MultiQC^15^. Basic quality assessments included: Phred scores, per sequence and per base quality score, GC contents, overrepresented k-mers, duplicated reads and presence of adaptors were re-checked. To identify global tendencies in the quality metrics output from MultiQC shows the quality across SC and OM samples (Fig. 1).

### Reads mapping and counts

**Fig. 2:**
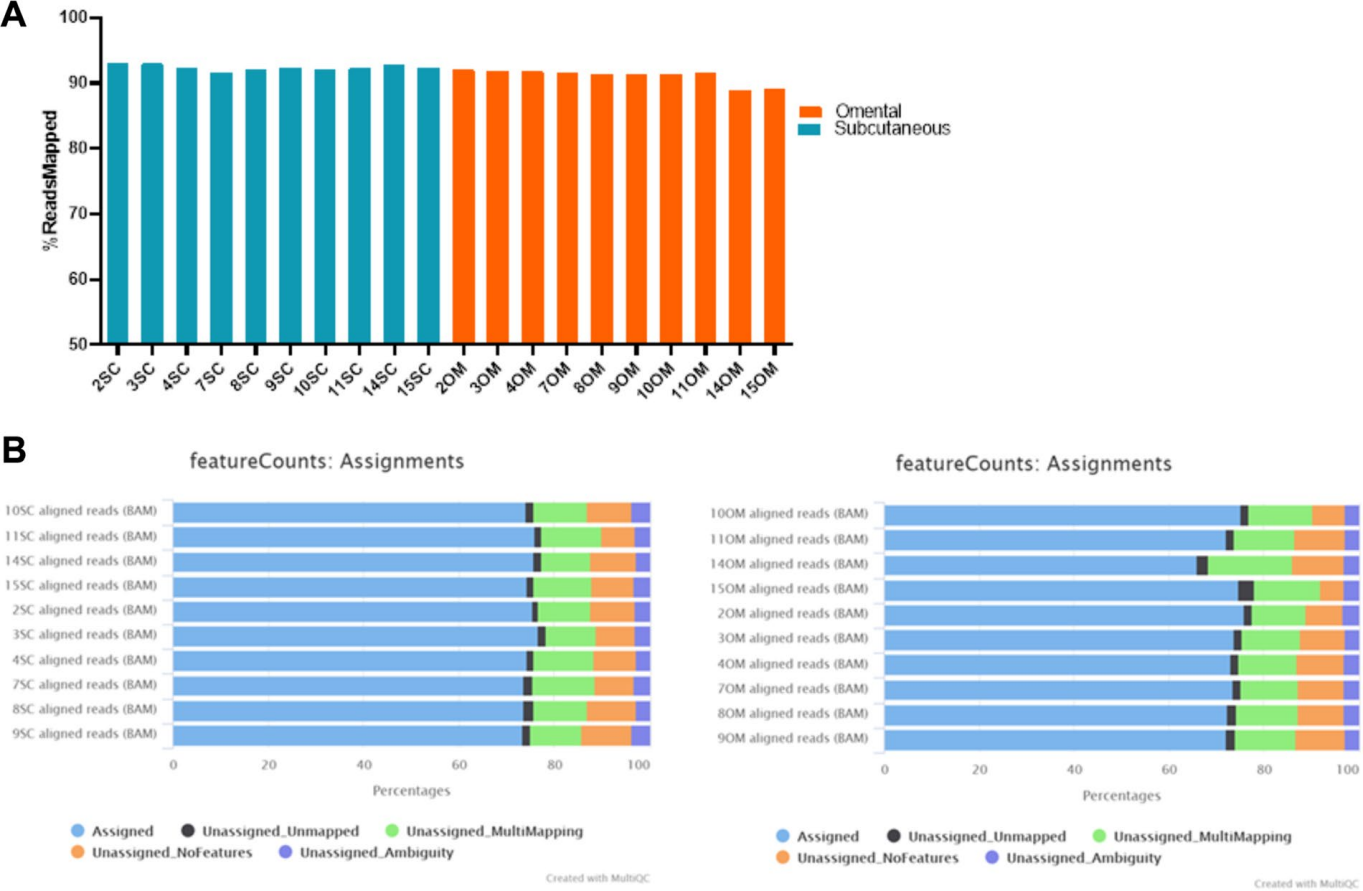
Mapping summary and counts assignment for each omental and subcutaneous adipose tissue sample collected from patients with gestational diabetes and matching controls. (A) Percentage of reads mapped to the (GRCh37/hg19). (B) Percentage of read counts assigned for Subcutaneous samples (left panel) and Omental (right panel).

After quality check, reads were mapped to the *Homo sapiens* reference genome (GRCh37/hg19) using HISAT 2.1.0^16^. BAM files obtained were sorted using SAMtools^17^ in the High Performance Computing at the Institute for Cyber-Enabled Research (ICER), Michigan State University. Mapping results are summarized in (Fig. 2A). The average mapping ratio with the reference genome was 91.8%. Next, featureCounts v.2.0.1^18^ was used to summarize the number of raw reads (Fig. 2B). On average 35,9 millions of reads (73,8%) were assigned to coding genes.

### Differential expression analysis in tissue-specific profiles

**Fig. 3:**
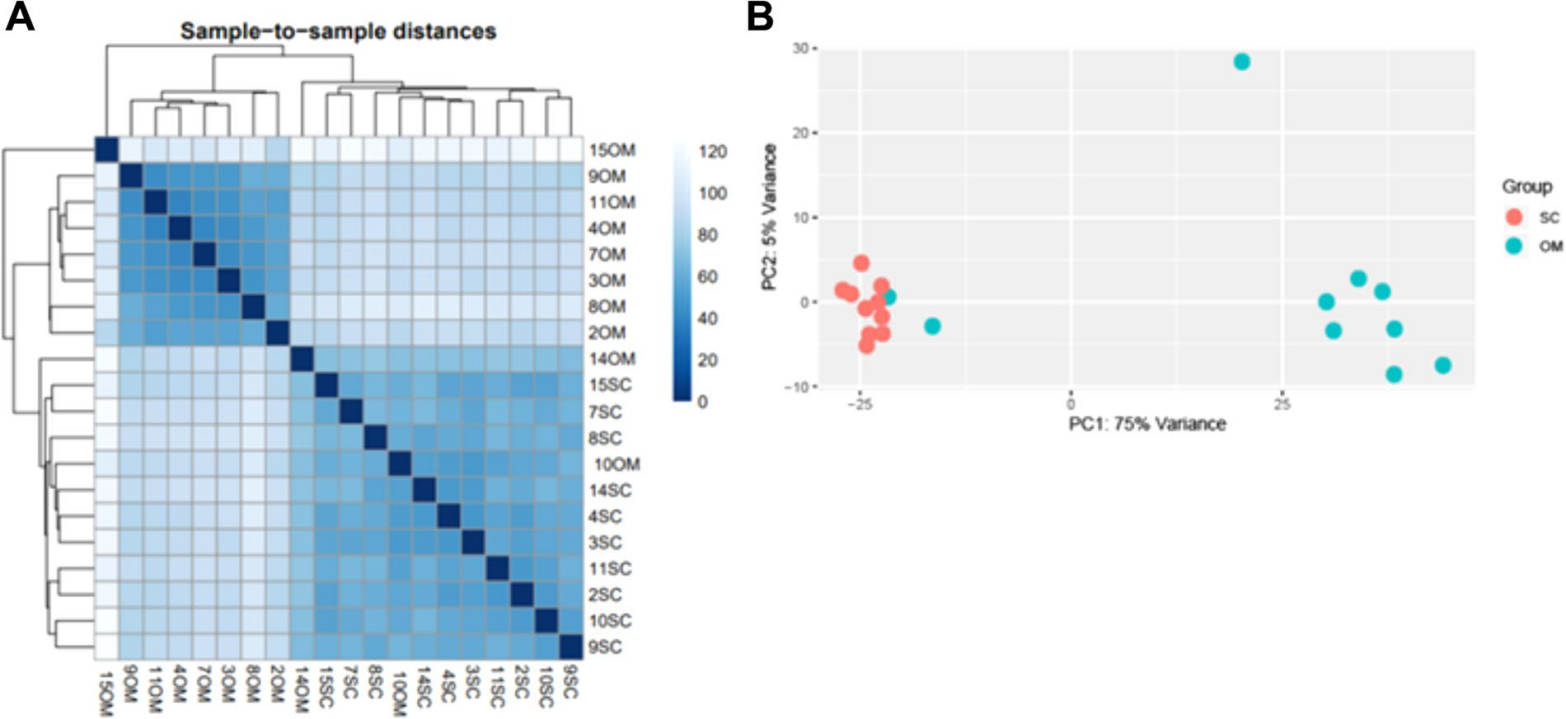
Tissue expression profile summary (**A**) Euclidean sample-to-sample distances. Samples were clustered using hierarchical clustering analysis, and dendrograms represent the clustering results. The heatmap illustrates the pairwise distances between the indicated samples. (**B**) PCA illustrates the cluster between subcutaneous (SC) and omental (OM) samples.

For differential expression analysis purposes, data counts were normalized through DESEQ. 2.0 negative binomial distribution model^19^. Sample variance was established using principal component analysis (PCA) plotting and hierarchical clustering (complete linkage method) using the Euclidean distances between samples (Fig. 3A). Samples from the same anatomic region clustered together indicating their expression profile is highly specific in both tissues (Fig. 3B**)**.

**Fig. 4:**
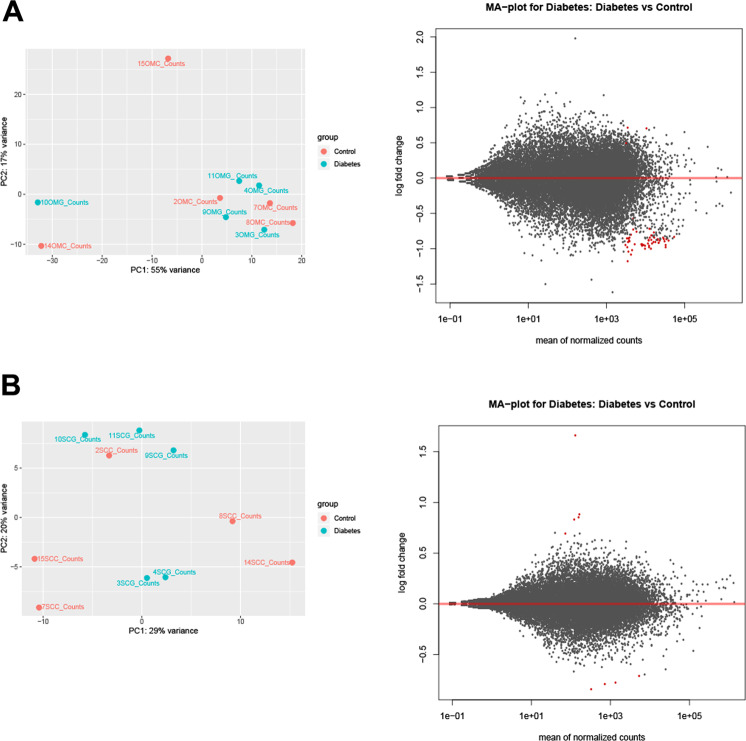
Transcriptomic expression profile in subcutaneous (SC) and omental (OM) adipose tissues from patients with gestational diabetes and matching controls. (**A**) PCA illustrates the cluster between OM samples from gestational diabetes patients (OMG) and thoses collected from matching controls (OMC). The MA plot shows the changes in gene expression of OM adipose tissue in gestational diabetes patients. (**B**) PCA illustrates the cluster between SC samples from gestational diabetes patients (SCG)and matching controls (SCC). MA plot shows the changes in gene expression of SC adipose tissue in gestational diabetes patients.

Differential Expressed Genes DEGs were determined by paired comparison between controls (OMC-SCC) and patients (OMG-SCG) in each specific tissue as follow OMG-OMC and SCG- SCC. For each comparison, the variance and gene expression changes between patients and control were established by DESEQ. 2. Genes with fold changes > 1 and FDR < 0.05 were defined as DEGs and captured for analysis (Fig.  4A-B), list of DEGs is available in (Supplemental Table 2^20^).

### Enrichment Analysis GSEA

**Fig. 5:**
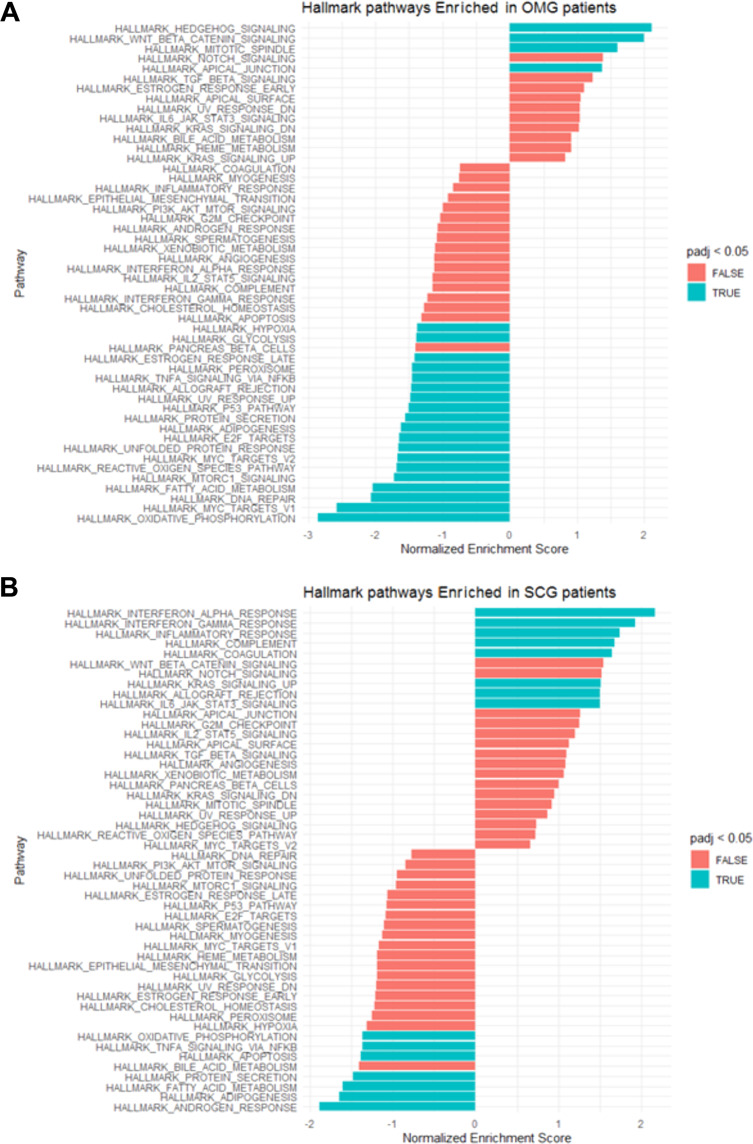
Signature enrichment in Omental and Subcutaneous tissue from Diabetes gestational patients (**A**) Omental (**B**) Subcutaneous. GSEA shows in the y axis 50 hallmark categories according to the Molecular signature Database and its enrichment in the profile expression of both tissues, in x axis Normalized Enriched Score (NES). Processes in red are not differentially enriched while those in green are differentially enriched at a *p-adjusted value > 0.05*.

After differential expression, Gene Set Enrichment Analysis (GSEA) was performed using the fgsea library in-house implementation in R studio (1000 permutations, term size of 15, and maximum term size of 500) to assess enrichment signatures in the expression profiles^21^. The entire gene lists were pre-ranked based on the mean fold change and significance (p-value) of each gene. The analysis included the gene set from the Molecular Signatures Database (MSigDB) pathways “Hallmarks”. The significance of enrichment was set by Benjamini-Hochberg false-discovery rate (FDR p‐value < 0.05) **(**Fig. 5**)**.

## Data Availability

This study was supported in part through computational resources provided by the Institute for Cyber-Enabled Research at Michigan State University **(ICER)**. The following software was used to perform quality and expression analyses of the dataset: 1. FastQC v0.72 https://www.bioinformatics.babraham.ac.uk/projects/fastqc/. 2. MultiQC v1.9 https://multiqc.info/. 3. HISAT2 v2.2.1 http://daehwankimlab.github.io/hisat2/. 4. SAMtools v1.9 http://www.htslib.org/. 5. featureCounts v2.0.1 https://www.rdocumentation.org/packages/Rsubread/versions/1.22.2/topics/featureCounts. 6. DESEQ v2.11.40.6 https://bioconductor.org/packages/release/bioc/html/DESeq2.html. 7. R v3.6.3.
